# Fluorescence Guided Raman Spectroscopy enables the training of robust support vector machines for the detection of tumour marker proteins

**DOI:** 10.1038/s41598-025-08425-0

**Published:** 2025-07-03

**Authors:** Johannes Reifenrath, Benjamin Gardner, Alexander Gigler, Friederike Liesche-Starnecker, Suzy Eldershaw, Nick Stone, Jürgen Schlegel

**Affiliations:** 1 Department of Neuropathology, Institute of Pathology, School of Medicine and Health, Technical University of Munich, Munich, Germany; 2 Biomedical Physics, University of Exeter, Physics and Astronomy, Exeter, UK; 3 Department of Earth & Environmental Sciences, Section Crystallography, Ludwig-Maximilians-Universität München, Munich, Germany; 4 Digital Industries, Center of Services – Portfolio & Strategy, Digital Industries, Siemens (Germany), Germany; 5 Medical Faculty , University of Augsburg , Augsburg, Pathology Germany

**Keywords:** Confocal Raman spectroscopy, Fluorescence microscopy, Classification, Supervised machine learning, Glioblastoma, Molecular diagnostics, Biophotonics, Confocal microscopy, Wide-field fluorescence microscopy, Raman spectroscopy, Preclinical research, CNS cancer

## Abstract

Raman spectroscopy provides comprehensive biochemical information on a sample’s composition, yet it is often used to analyze aggregated spectra rather than specific shifts. We introduce Fluorescence Guided Raman Spectroscopy (FGRS) as a methodology enabling the isolation of proteins’ spectral signatures and the training of classifiers that generalize across cell lines. We demonstrate the utility of this approach using connexin 43, a marker protein of glioblastoma tumour microtubes. By screening eGFP, sodium fluorescein, and mTagBFP2 for their compatibility with a Raman system operating at 532 nm, we selected mTagBFP2 as the most Raman-compatible fluorophore, whereas the other fluorophores emitting near 532 nm caused spectral interference. mTagBFP2 was cloned into a connexin 43 expression vector, allowing fluorescent tracking and Raman interrogation with subsequent peak identification and correlation to an I-TASSER protein prediction model. We then trained two support vector machines (SVMs) for the classification of cells based on their connexin 43 content and highlighted the impact of different spectral ranges (full spectrum vs. most significant Raman shifts) on specificity and sensitivity in glioblastoma target cell lines. Connexin 43 expression led to a loss of the peaks at 600, 1253, and 1401 cm⁻¹, consistent with an increased α-helical content as predicted by I-TASSER. SVMs achieved up to 79% accuracy on unseen glioblastoma lines, with full-spectrum models reaching 98.7% sensitivity. Thus, FGRS enables the spectral isolation of tumour marker proteins and the development of robust classifiers across cell lines. By focusing on key Raman shifts, this method holds the potential to improve diagnostic accuracy and sensitivity, offering a customizable tool for tumour detection.

**Supplementary Information:**

The online version contains supplementary material available at 10.1038/s41598-025-08425-0.

## Introduction

Genetic information can significantly influence a patient’s therapy regimen based on individual biomolecular traits and, therefore, the prognosis^1,2^. However, some patients do not show clinical benefit from genetically targeted therapies because of post-translational modifications and intratumour heterogeneity^3–5^. In fact, for some cancer entities, such as glioblastoma multiforme and pancreatic cancer, prognosis has changed little over the past decades and remains low at 15–16 months^6,7^. In response, personalized medicine is striving to integrate comprehensive genetic, proteomic, and lipidomic data into diagnosis and treatment options^5,8^. Yet the clinically available methods for obtaining such information, such as immunostaining, sequencing, and mass spectrometry, are often laborious and slow, as they require extensive sample preparation protocols^9,10^.

Raman spectroscopy (RS) offers a potential diagnostic shortcut. It is a label-free, non-destructive method that can extract comprehensive biomolecular information from cells with little to no sample preparation. Technically, spontaneous RS is based on the inelastic scattering of light, i.e., the change in a photon’s energy and, thus, its wavelength (Stokes shift) as it interacts with a molecule^11,12^. Stokes shifts are specific for each molecular vibration^11,13,14^. Therefore, the combination of the measured shifts provides a fingerprint of each of the molecules found in the sample volume. RS so delivers a biochemical fingerprint for each probe, containing unique features for pre- and posttranscriptional nucleic acids, lipids, proteins, and other molecules^12,14^.

The diagnostic properties of RS can be used to distinguish neoplastic from healthy tissue in oncology^15^ and surgery^16^. Many of the respective classifiers require the entirety of a Raman spectrum as input and return their results based on the overall compositional differences between malignant and healthy tissue^16,17^. To our knowledge, there is a relative paucity of classifiers trained on the unique spectral shifts raised by a specific protein.

Raman sampling of distinct intracellular structures is challenging due to both the complex localization of the structures of interest as well as the sophisticated interpretation of the data. It should be noted that Raman spectra contain a mixture of signals from all of the molecules in the sample volume. This is both a strength and a weakness, as specific molecules can be harder to identify from individual peaks. Therefore, some authors have made their molecules of interest amenable to Raman interrogation by selective isolation from bodily fluids^18^ or synthetic production^19^. Also, spectroscopically distinct labels, such as deuterium- or alkyne tags, have been used^20,21^ to provide a locational clue. However, these methods are limited in their applicability because many molecules cannot be easily isolated from body fluids, synthesized, or linked to alkyne or deuterium tags.

To address this gap, we present Fluorescence Guided Raman Spectroscopy (FGRS) as a method to selectively obtain the spectroscopic features of a protein in its native cellular environment. By fusing a blue-shifted fluorescence protein with a protein of interest, we can locate the protein, collect its spectral fingerprint, and train classifiers for its detection.

We prove the feasibility of the proposed method for Connexin 43 (Cx43) C-terminally fused to mTag blue fluorescent protein 2 (mTagBFP2). The latter is a blue-shifted derivative of the GFP-like protein TagRFP^22,23^, while Cx43 is a four-pass transmembrane protein with an intracellular N- and C-terminus and a prominent alpha-helical structure^24^. En route to the membrane, Cx43 travels through the endoplasmic reticulum (ER) and the Golgi-Apparatus, where monomers are phosphorylated and form hexameric connexons, which then connect to another cell’s connexon in the membrane^25,26^. The C-terminus is available for interactions with other proteins and has many functions, including regulatory ones for channel opening probability, gene expression, proliferation, and migration^27^. In experimental settings, connexins’ C-termini have been used for tagging with fluorescent proteins with no functional impairment of expression or gap junction formation^28,29^. We thus hypothesize that the introduction of mTagBFP2 would not impede the expression of Cx43 for subsequent Raman sampling.

Cx43 is of particular interest in neurooncology, where it is a functional protein in glioblastoma (GBM) tumour microtubes, connecting cells to a chemotherapy-resistant cellular network with a shared calcium homeostasis^30,31^. It is suggested that the intraoperative detection of tumour microtubes could enhance the resection of GBM stem cell networks and improve adjuvant therapy response to chemoradiation^31^.

We believe Cx43 is ideally suited for FGRS, as it is (a) hard to synthesize due to its oligomeric structure, yet (b) of clinical importance. The objective of this study is to fluorescently tag Cx43 (Cx43-mTagBFP2), making it detectable for Raman interrogation in its native environment. Our first hypothesis is that the emission wavelength of mTagBFP2 does not obscure the Raman spectrum using a 532 nm laser and collected with a detection system at longer wavelengths. Second, we propose that we can identify discrete spectral alterations induced by the presence of Cx43 in cell lines. For this, we present the development of four classification algorithms to discern HEK293 cells with high and low Cx43 content. Finally, we verify our classifiers on four human GBM cell lines with different Cx43 expression levels.

## Results

### mTagBFP2 is a fluorophore compatible with Raman imaging at 532 nm

**Fig. 1:**
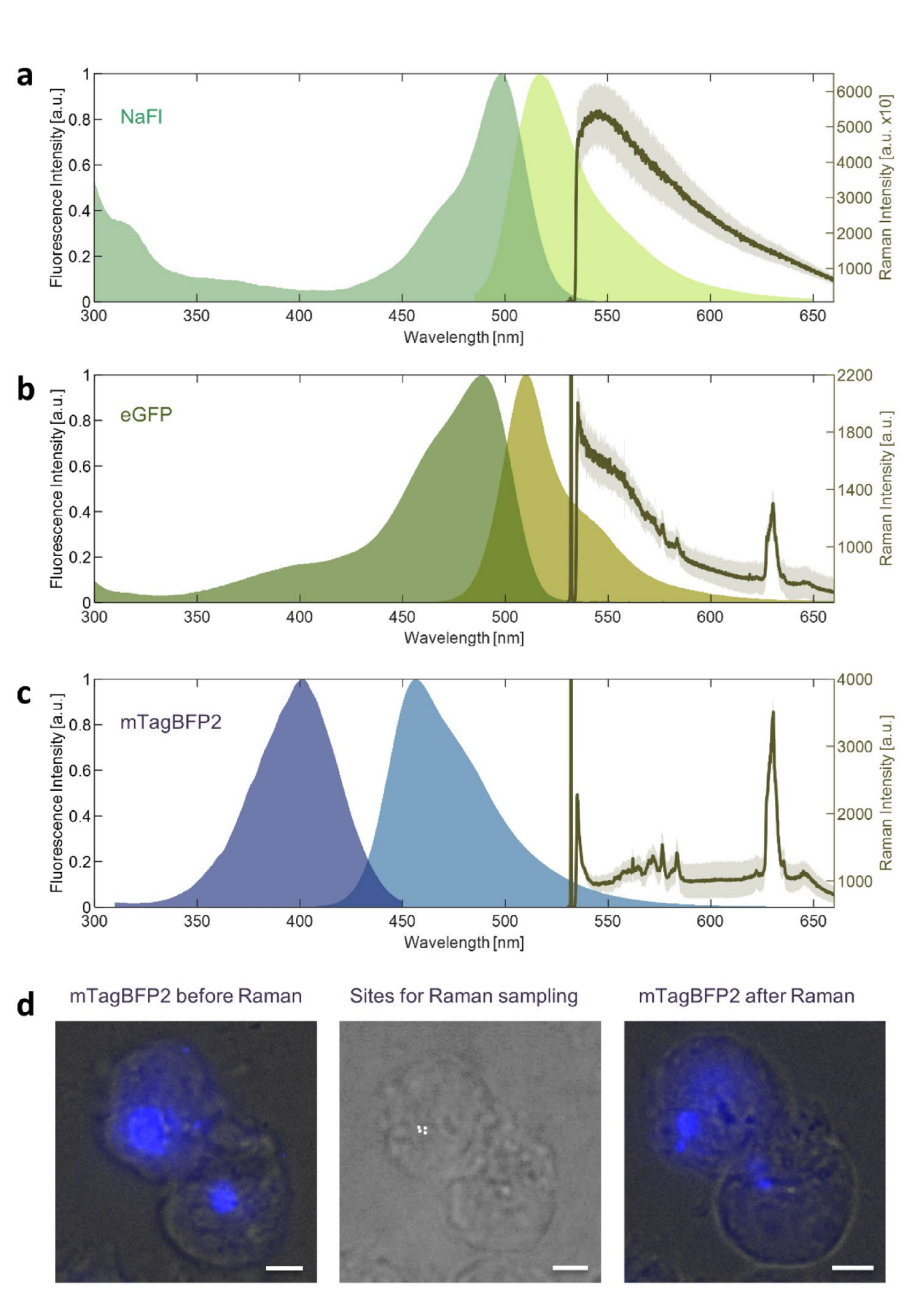
The effect of various fluorescence spectra on Raman sampling. (**a**) Sodium fluorescein (NaFl): excitation/emission $\:\approx\:$498 nm (dark green)/$\:\approx\:\:$517 nm (light green) and its Raman spectrum in a HEK293 cell (solid line) with the first standard deviation (shade). (**b**) eGFP: excitation/emission 488 nm (dark green)/507 nm (ocher) and its Raman spectrum in a HEK293 cell (solid line) with the first standard deviation (shade). (**c**) mTagBFP2: excitation/emission 405 nm (dark blue)/454 nm (light blue) and the Raman spectrum of mTagBFP2-Cx43 in a HEK293 cell (solid line) with the first standard deviation (shade). (a-c) Raman spectra are not baselined. Spectra were obtained from FPbase^35^ (d-f) HEK293 cells expressing mTagBFP2-Cx43. White bar $\:\approx\:\:$5 μm (**d**) Fluorescence imaging before (left) and after (right) Raman sampling on a fluorescent microscope shows no significant loss in fluorescence. The sites for Raman sampling can be identified in correlating brightfield imaging on the Raman microscope (middle).

We screened three fluorophores for their spectral interference with a Raman system using a 532 nm excitation laser. Of the tested fluorophores, sodium fluorescein (NaFl; 465–490/500–550 nm)^32,33^ was closest to the Raman laser and the only one to completely obscure the Raman signal (Fig. 1a). The enhanced green fluorescent protein (eGFP) (488/509 nm)^34^ showed a significant baseline elevation of the Raman signal (Fig. 1b). The shape of the baseline shift of the Raman signal resembled the emission spectrum of eGFP with an initial and terminal steep slope and a mid-range slurring. mTagBFP2 (399/454 nm)^23^ showed no significant fluorescence leaking into the Raman signal (Fig. 1c). Peaks in the fingerprint region were clearly distinguishable. To exclude the possibility of photobleaching before the Raman image acquisition we checked the fluorescence of mTagBFP2 before and after interrogation but detected no significant loss in intensity (Fig. 1d). In general, the lower the fluorophore’s emission wavelength, the further it was from the Raman spectral range and, therefore, the lower its interference with the Raman signal.

### Cx43-mTagBFP2 is reliably expressed and located in HEK293 cells for Raman spectroscopy

#### The secondary structure of Cx43 and mTagBFP2 is retained in the fusion protein

**Fig. 2:**
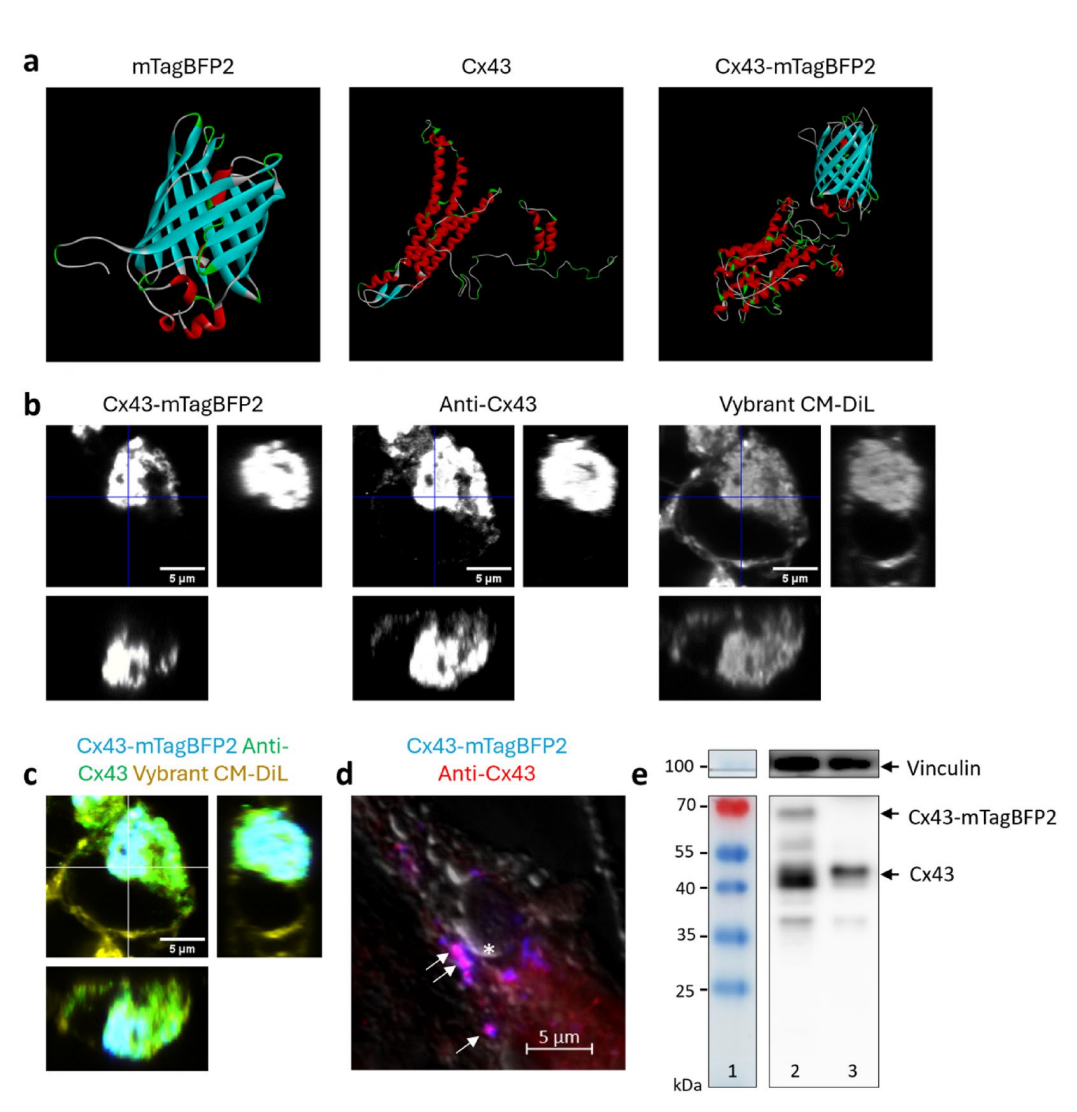
Expression of Cx43-mTagBFP2 in HEK293 cells. (**a**) Predicted protein models for mTagBFP2, Cx43, and Cx43-mTagBFP2 (light blue: β-pleated sheets, red: α-helices). (**b**,** c**) Confocal fluorescence images of HEK293 expressing Cx43-mTagBFP2 show the colocalization of Cx43-mTagBFP2 and the anti-Cx43 antibody. Square image: view of the XY-plane, right image: orthogonal view of the YZ-plane along the vertical blue or white line, bottom image: orthogonal view of the XZ-plane along the horizontal blue or white line. **(d**) Widefield epifluorescence image showing the colocalization of Cx43-mTagBFP2 (blue) and anti-Cx43 antibody (red) at the characteristic membranous locations. (**e**) Cropped western immunoblot with an anti-Cx43 antibody (bottom) and an anti-vinculin antibody (loading control, top) (1: blot ladder with molecular weight indicated on the left, 2: HEK293 cells expressing Cx43-mTagBFP2, 3: HEK293 wildtype). The fusion protein is observed at around 70 kDa in the transfected cells, but not in the wildtype cells. Both cell types also show bands at around 43 kDa. Exposure time: 40 s. Uncropped blot in Supplementary Material S5

Next, we explored whether a fusion protein composed of Cx43 and mTagBFP2 would retain the secondary structure of its individual constituents. For this, we employed the I-TASSER-server to model each protein’s structure. I-TASSER is an online server predicting the structure of a protein based on its amino acid sequence by iterative threading assembly simulations^36,37^. The top threading model for the predicted structure of mTagBFP2 was a mutant of the fluorescent protein mKate S158A, which was previously characterized by synchrotron radiation^38^. It showed a 92% sequential homology and resembled the typical β-barrel structure of fluorescent proteins^22,23^ (Fig. 2a; Table 1). Accordingly, the confidence score for the predicted secondary model was high at 7.15 out of 9.

The structure of Cx43 was less well-defined and modelled on sequences from three previously defined proteins. The N-terminal region was predominantly modelled on connexin 32 and a C-terminally truncated version of Cx43, exhibiting an α-helical structure (Fig. 2a; Table 1). These two templates covered 54% and 53% of the complete Cx43 with a respective sequence identity of 48% and 100%. The majority of the protein, including the C-terminus, was identified as a random coil^39^ (Table 1). The template covered approximately 34% of the human amino acid chain C-terminally with a sequence identity of 96%. The average confidence score for the predicted secondary structure was 6.51 out of 9 and considerably higher in the transmembrane helical structures (confidence score of 9).

The fusion molecule Cx43-mTagBFP2 largely retained the secondary and tertiary structure of its individual components (Fig. 2a). The number of alpha-helices with a minimum length of five amino acids for the Cx43-part remained unchanged at eight. The index of the initial and terminal amino acid for each alpha-helix in the Cx43-part varied by a maximum of two. For the mTagBFP2-part we observed 11 beta sheets in lifeact7-mTagBFP2 and one additional beta sheet of six amino acids in the fusion protein. The indices of the 11 mutual sheets varied by no more than two. For the linking amino acid sequence, I-TASSER predicted predominantly a random coil. The overall confidence score in the secondary structure of the fusion protein was 6.22 out of 9. For the linking amino acid sequence, I-TASSER predicted a random coil. The overall confidence score in the secondary structure for the fusion molecule was 6.22 out of 9.

**Table 1:** Results of I-TASSER protein model prediction for secondary Structure.

	mTagBFP2		Cx43		Cx43-mTagBFP2	
α-helix	19/237	(8.0%)	152/382	(39.8%)	169/636	(26.6%)
β-strand	115/237	(48.5%)	8/382	(2.1%)	130/636	(20.4%)
Coil	103/237	(43.5%)	222/382	(58.1%)	337/636	(53.0%)
total	237	(100.0%)	282	(100.0%)	636	(100.0%)

The top three rows list the secondary structure with their absolute and relative number of amino acids for mTagBFP2, Cx43, and the fusion protein Cx43-mTagBFP2 as predicted by the I-TASSER prediction model. The bottom row indicates the total number of amino acids for each protein.

#### Intracellular accumulations of Cx43-mTagBFP2 are accessible for Raman imaging

In FGRS, fluorescence imaging is a prerequisite for subsequent Raman sampling. We transiently transfected the expression vector for Cx43-mTagBFP2 into HEK293 cells. Figure 2b and c show that a polyclonal anti-Cx43 staining overlaps with the fluorescent signal from the fusion protein Cx43-mTagBFP2, indicating a positive transfection (Fig. 2b, c). Cx43 could be observed at the cellular membrane (Fig. 2d). We also noted a strong fluorescence peak inside the cell, which in a Vybrant CM-DiI membrane stain colocalized with other intracellular membranes (Fig. 2b, c). This location likely corresponds to the physiologic collection of Cx43 in the endoplasmic reticulum (ER) or Golgi apparatus, as the protein is oligomerized en route to the membrane^40^. Since these deposits would have the greatest intracellular concentration of the fusion protein while being structurally similar to membranous Cx43, we selected these locations for the subsequent Raman measurements.

#### Western blot of Cx43-mTagBFP2

In a western immunoblot, there was a strong band at 70 kDa (Fig. 2e), approximating the sum of mTagBFP2 (26.7 kDa)^35^ and Cx43 (43 kDa)^41^. We also observed bands at around 43 kDa in transfected and in wildtype cells.

### Raman imaging

#### Specific Raman shifts identified for Cx43-mTagBFP2 and wildtype HEK293 cells

**Fig. 3:**
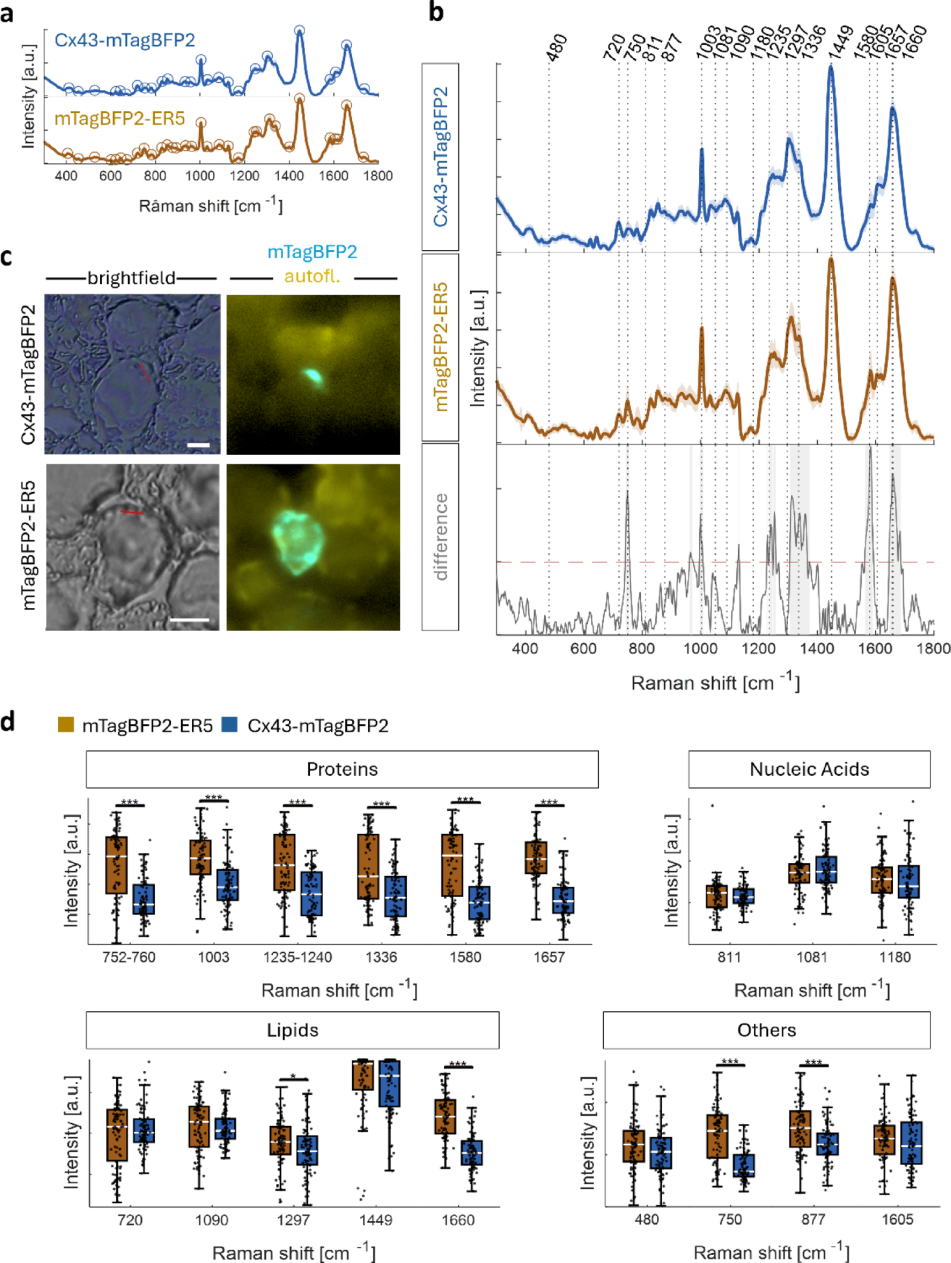
Comparison of Raman spectra of Cx43-mTagBFP2 and mTagBFP2-ER5. (**a**) The average spectra of Cx43-mTagBFP2 and the control mTagBFP2-ER5 show distinct spectral peaks at the significance level of *p* ≥ 0.01. (**b**) Overview of the average Raman intensity (solid line) and its first standard deviation (shade) at known Raman shifts for Cx43-mTagBFP2, mTagBFP2-ER5, and their absolute difference. Raman shifts where differences exceeded the double standard deviation (dashed red line) are shaded in light grey. (**c**) Correlation of the brightfield and fluorescent images allows colocalization of the regions of interest for measurement (red line). *autofl.* indicates imaging based on green autofluorescence. White bar: $\:\approx\:$5 μm (**d**) Comparison of the peak intensity measured at known Raman shifts identifies significant changes for proteins. (*n* = 95, Bars extend between 25th and 75th percentile. White line indicates the median. Black dots are individual data points. Wilcoxon rank sum test * *p* < 0.05, **p$\:{\le\:10}^{2}$, p$\:{\le\:10}^{3})$.

The second step in FGRS is Raman sampling. We first assessed spectral changes between the Raman spectra of the fusion protein Cx43-mTagBFP2 and the control fluorescent protein mTagBFP2-ER5 (Fig. 3a-c) expressed in HEK293 cells. For each protein, we identified representative Raman shifts Table (2) by their peak location and peak intensity in the fingerprint region (600–1800 cm^−1^).

**Table 2:** Assignment of Raman Peaks.

Shift [cm^–1^]	Assignment				References
	Protein	Nucleic Acids	Lipids	Others	
**480**				Glycogen	^64,71^
**720**		C-N stretching Adenosine	C-N stretching		^72^
**746/750**				Cytochrome C	^55,64,73^
**750/752–760**	Ring breathing in Tryptophan				^72,74,75^
**811**		RNA: C5 -O-P-O-C3 phosphodiester bonds stretching			^72^
**877**			C-C-N+ symmetric stretching	Carbohydrate: C-O-H ring	^72^
**1003**	Phenylalanine ring-breathing				^64,71^
**1081**		PO2 − symmetric stretching			^76^
**1090/1095**			Cytosine, Guanine, Adenosine		^17,72^
**1180**		Cytosine, Guanine, Adenosine			^72^
**1235–1240**	Peak: β-pleated sheet; absence: α-helix				^54^
**1295/1297/1300**			CH^2^ vibrations		^17,64,71^
**1337**	In the Amide III region				^71^
**1449/1452**	CH_2_ / CH_3_ vibrations		CH_2_ / CH_3_ vibrations		^71,76^
**1580**	High wavenumber end of Amide II (1550-1580)				^77^
**1582**				Cytochrome C	^55^
**1605**				Retinoic Acid	^78^
**1654–1655**	Amide I: C=O stretching mode, peptide linkage				^71,72,76^
**1660**			C-C stretching		^64,72^

Raman shifts and their respective chemical groups for proteins, nucleic acids, lipids, and other biological substances.

Peaks were identified by their prominence in comparison to neighbouring peaks using a prominence-value of 0.01. We detected a small number of peaks in the control fluorescent protein that were not present in the Cx43-mTagBFP2 cells (Fig. 3a, supplementary material S1). At 600 cm^−1^ and 1401 cm^−1^, we identified peaks with no clear correlation in the Cx43-mTagBFP2 sample. At 876 cm^−1^, 892 cm^−1^, and 1618 cm^−1^, both samples showed a corresponding peak formation, but the Cx43 samples did not exceed the prominence level. Further, the control fluorescent protein exhibited a prominent peak in the Amide III region at approx. 1253 cm^−1^.

Spectral intensities were generally significantly different for Raman shifts associated with proteins, but less frequently so for shifts associated with nucleic acids or lipids. Raman shifts associated with proteins showed the most significant spectral intensity differences (Fig. 3d). We computed the effect size as a quantitative measure of the magnitude of the statistical difference^42^. It was greatest for the 1657 cm^−1^ Raman shift (d = 1.98), followed by the Raman shifts at 1580 cm^−1^ (d = 1.30), 752–760 cm^−1^ (d = 1.29), 1235–1240 cm^−1^ (d = 1.05), and at 1003 cm^−1^ (d = 1.05). The smallest effect size was observed at 1336 cm^−1^ (d = 0.91). In contrast, the Raman shifts for glycogen and all-trans-retinol at 480 cm^−1^ and 1605 cm^−1^ did not result in significant intensity differences (Fig. 3d). However, the Raman shifts for lipids and carbohydrates at 877 cm^−1^ and cytochrome c at 750 cm^−1^ and 1582 cm^−1^ exhibited significant intensity changes (d_877_ = 0.72; d_750_ = 1.44; d_1582_ = 1.39). For lipids, the Raman shifts at 720 cm^−1^, 1090 cm^−1^, and 1449 cm^−1^ were not significantly different (Fig. 3d), yet there were significant intensity differences for the Raman shifts associated with lipids at 1297 cm^−1^ and 1660 cm-1 (d_1297_ = 0.27; d_1660_ = 1.78). For nucleic acids, we detected no significant intensity variations between Cx43-mTagBFP2 and mTagBFP2 at 720 cm^−1^, 811 cm^−1^, 1081 cm^−1^, and 1180 cm^−1^ (Fig. 3d).

#### Distinct Raman shifts identified in four human glioblastoma wildtype cell lines

**Fig. 4:**
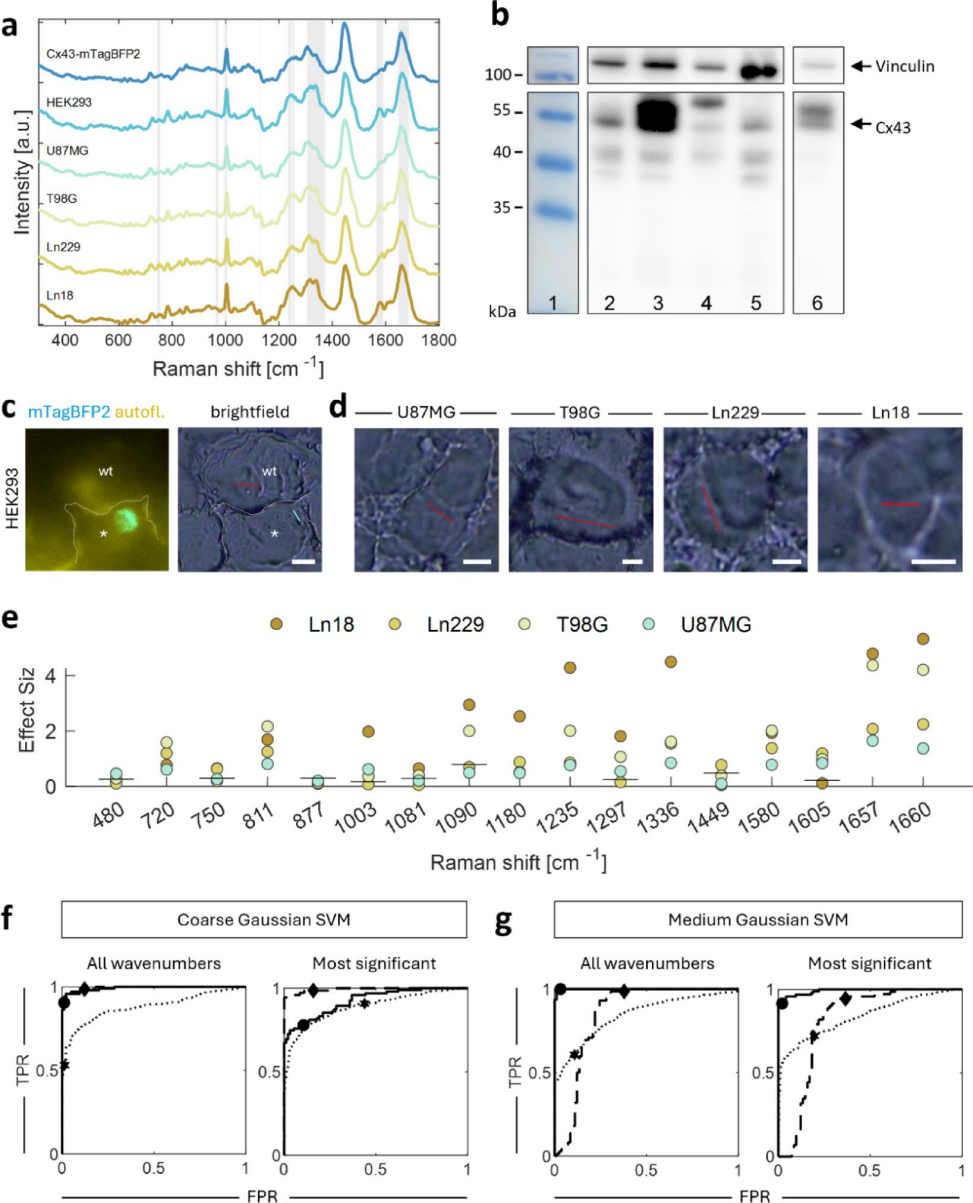
Comparison and Classification of Raman spectra of human GBM wildtype cell lines. (**a**) Average spectrum (solid line) and first standard deviation (shade) for the indicated cell lines. Raman shifts where the difference exceeded the double standard deviation are shaded in light grey. (**b**) Cropped western immunoblot with an anti-Cx43 antibody (bottom) and an anti-vinculin antibody (loading control, top). (1: blot ladder with molecular weight indicated to the left, 2: T98G, 3: U87MG, 4: Ln229, 5: Ln18. Exposure time: 3 min. 6: Lane 3 at lower exposure time (7.5 s)). Uncropped blots in Supplementary Material S6. (**c**) Epifluorescent and correlating brightfield images of a HEK293 cell expressing Cx43-mTagBFP2 (*, aprox. cell borders denoted by dotted line) and an adjacent wildtype cell (wt). Raman sampling for Cx43-mTagBFP2 was conducted along the blue line; for wildtype, along the red line. (**d**) Brightfield images of probed wildtype cells. White bar: $\:\approx\:$5 μm. (**e**) Effect sizes of the intensity differences at the indicated Raman shifts between HEK293 cells expressing Cx43-mTagBFP2 and the indicated cell lines. Ln18 (brown), Ln229 (dark ocher), T98G (light ocher), U87MG (turquoise). The horizontal lines represent the significance levels. Dots centered below the line do not reach the significance level. (**f**,**g**) ROC curves of the coarse and medium Gaussian SVMs using all Raman shifts and the most significant Raman shifts, i.e., those where the intensity difference exceeded the double standard deviation. TPR: true positive rate FPR: false positive rate.

We then analysed the Raman spectra of the human GBM cell lines U87MG, T98G, Ln229, and Ln18 (Fig. 4a-d). Using a prominence-value of 0.05, we detected peaks shared by all cell lines at approximately 720 cm^−1^, 850 cm^−1^, 938 cm^−1^, 1004 cm^−1^, 1449 cm^−1^, and 1660 cm^−1^ (supplementary material S2). Of note, Cx43-mTagBFP2 and U87MG could be identified by a mutual loss of the peaks at around 1250 cm^−1^ and 1580 cm^−1^, whereas all other cell lines showed a prominent peak. Cx43-mTagBFP2 was also the only sample to show no peak at around 784 cm^−1^. We then compared the spectra of the GBM wildtype cell lines to the spectra of Cx43-mTagBFP2 by calculating the statistical effect size for the previously discussed Raman shifts for proteins, lipids, nucleic acids, and other substances. The average effect size across all Raman shifts was the smallest for the difference between Cx43-mTagBFP2 and U87MG cells at 0.64 (Fig. 4e). We observed the single smallest effect sizes at 1449 cm^−1^ and at 877 cm^−1^. In contrast, we calculated the largest average effect size for the difference between Cx43-mTagBFP2 and Ln18 cells.

U87MG is known to express the highest level of endogenous Cx43^43^. We confirmed this for our U87MG cells in a western immunoblot with a polyclonal anti-hCx43-antibody (Fig. 4b). We observed prominent bands at approx. 43 kDa for U87MG cells, and only weak bands for T98G, Ln229, and Ln18 cells. We thus classified U87MG as “high Cx43 content” and all other cell lines as “low Cx43 content”.

### Classification of cell lines based on their Cx43 content

#### Training support vector machine classifiers with high accuracies in HEK293 cells

Based on these findings, we concluded that spectral intensity differences exist that can be employed to train classification algorithms to discriminate between cells of high and low Cx43 content. We trained a medium and coarse Gaussian SVM on HEK293 cells transfected with Cx43-mTagBFP2 or the control fluorescent protein mTagBFP2-ER5.

First, we trained the classifiers with on the full spectral range of the acquired data (571 wavenumbers), which resulted in a remarkable training accuracy of 94.7% for the coarse and 98.4% for the medium Gaussian SVM classifier (Table 2). Second, to increase robustness when applied to other cell lines, we trained the classifiers only on the Raman shifts where the spectral intensity difference exceeded the double standard deviation. This included primarily Raman shifts associated with proteins and the shift at around 1580 cm^−1^. The coarse and medium Gaussian SVM achieved training accuracies of 83.7% and 94.7%, respectively (Table 3, supplementary materials S3/S4).

#### Testing the SVMs by distinguishing HEK-Cx43-mTagBFP2 from wildtype HEK293-cells

The classifiers were then used to discern positively transfected HEK293 cells (high Cx43) from adjacent HEK293 wildtype cells (low Cx43, Fig. 4a, c). Coarse Gaussian SVMs performed significantly better than their medium Gaussian counterparts (Table 3 Testing 1). The coarse Gaussian SVM’s accuracy trained on all wavenumbers was comparable to the training accuracy (92.9%), while the one trained on only the most important Raman shifts improved to 90.9%, mainly because of the high specificity of 98%. Medium SVMs showed a reduced accuracy of 79.2% when using all wavenumbers and 77.9% when using only the preselected shifts. The superior performance of the coarse Gaussian SVMs can also be inferred from their rectangularized random operator characteristic (ROC) curve (Fig. 4f, g).

#### Testing the SVMs on four human GBM wildtype cell lines: number of Raman shifts allows optimizing for sensitivity or specificity

The coarse and medium SVMs trained on only the most significant Raman shifts showed higher testing accuracies (79.0% and 74.9%, resp.) and specificity (90.8% and 72.1%, resp.) than their counterparts trained on a complete spectrum (Fig. 4f, g; Table 2 Testing 2). Inversely, the SVMs trained on all wavenumbers were more sensitive than SVMs trained only on the most significant Raman shifts.

The coarse Gaussian SVMs showed the highest overall specificity (90.8% for the most significant Raman shifts) and sensitivity (98.7% for all wavenumbers), but at the expense of the other parameter dropping below 60%. For the medium Gaussian SVM the significance and specificity differ less from each other.

**Table 3:** Classification Results.

	Accuracy [%]	AUC	Sensitivity [%]	Specificity [%]	Accuracy [%]	AUC	Sensitivity [%]	Specificity [%]
Coarse Gaussian SVM								
Data Used	All Wavenumbers				Raman Shifts Greater Double Standard Deviation			
Training	94.7	0.99	98.9	90.5	83.7	0.92	89.5	77.9
Testing 1	92.9	0.99	87.8	98.6	90.9	0.99	84.1	98.6
Testing 2	68.6	0.88	98.7	53.1	79.0	0.89	56.2	90.8
Medium Gaussian SVM								
Data Used	All Wavenumbers				Raman Shifts Greater Double Standard Deviation			
Training	98.4	1.00	96.8	100	94.7	0.99	97.9	91.6
Testing 1	79.2	0.85	62.2	98.6	77.9	0.81	63.4	94.4
Testing 2	70.4	0.84	89.2	60.7	74.9	0.84	80.4	72.1

Table 3 summarizes the classification results. Training: high-Cx43 content cells were HEK293 expressing C43-mTagBFP2; low-C43 were HEK293 expressing mTagBFP2-ER5. Testing 1: high-Cx43 same as for training; low-Cx4 were HEK293 wildtype cells. Testing 2: high-Cx43 content cells were U87MG cells; low-content T98G, Ln229, and Ln18. For “All wavenumbers” we computed 571 Raman shifts; for “Raman Shifts Greater Double Standard Deviation” 82 spectra were used. AUC: area under the curve.

## Discussion

Molecular pathologic analysis of tumours can identify a plethora of marker proteins and therapeutic targets, but it is an inherently slow and work-intensive process^9^. In contrast, RS can deliver fast biochemical information on a sample^44^. However, current Raman classifiers are generally trained independently from proteomic and genomic discoveries because there is no versatile method for the collection of a specific protein’s Raman spectrum in its native environment. Therefore, the objective of study was to address this gap by developing FGRS to obtain the spectra of proteins. We identified mTagBFP2 as a fluorescent protein compatible with standard blue fluorescence filters and a Raman system operating at 532 nm. We obtained the spectrum of Cx43, a gap junction protein involved in resistant glioma networks, and trained four classification algorithms for its identification in glioblastoma cell lines.

We first fused Cx43 to mTagBFP2 and validated its expression by fluorescence microscopy, western blot, and an I-TASSER protein prediction model. We observed Cx43-mTagBFP2 at the physiological expression sites for Cx43, most importantly intracellularly and on the cell membrane^41^. The intracellular accumulation of Cx43-mTagBFP2 resembled previously described expression patterns of Cx43 as it passes through the ER and Golgi-apparatus^41,45^. This site was chosen for Raman sampling because (a) it was easily detectable and (b) it ensured a local maximum concentration of Cx43-mTagBFP2, yielding stronger and specific spectroscopic signals. According to the protein prediction model, Cx43 retains its characteristic alpha-helical structure. This was expected since fluorescently tagged connexins have been used for functional studies before^28,46^. Finally, we confirmed the molecular weight of Cx43-mTagBFP2 with a western immunoblot showing the fusion protein at the anticipated weight and in line with other studies^47^.

We then demonstrated that the spectral properties of mTagBFP2 (399/454 nm) and RS at 532 nm are sufficiently distinct to allow hybrid sampling with RS and fluorescence imaging. This merits special consideration, as overlapping fluorescence is the nemesis of spontaneous RS, because Raman signals are many orders of magnitude weaker than their fluorescent counterparts and can thus be obscured^48,49^. Fluorescence originates from photon absorption – an energetically relatively intense process - while Raman signals result from the inelastic scattering of light, an energetically less intense process^11,12^. Several groups used fluorescent proteins in Raman imaging before, but to our knowledge, this study reports the closest spectral proximity between a blue fluorescent protein’s emission wavelength and the Raman excitation laser. Chiu et al. al (2017) combined the blue and enhanced cyan fluorescent proteins (emission 445 nm and 476 nm) with a 532 nm Raman laser but used an anti-Stokes fluorescence detection scheme that allowed focusing on a blue shifted emission^50^. A greater spectral distance between fluorescence emission and the Raman laser (785 nm) was chosen by Yuan et al. (2018) for the sampling of enhanced cyan fluorescent protein^51^, while Huang et al. (2007) probed the green fluorophore Cyanine 3 with a 532 nm laser after a photobleaching step^52^. Alternatively, in their review of intraoperative Raman and fluorescence imaging, Lauwerends et al. (2022) suggest red-shifting the Raman laser past the fluorescence excitation wavelength into the high wavenumber region beyond 2400 cm.^1^^53^.

Next, we showed that a discrete set of spectral changes could be identified for Cx43 and used to train classifiers. We observed significant differences in the peak locations and intensities. In the Amide III band, the presence of an intense Raman shift at 1230–1240 cm^−1^ has been reported as one of the most characteristic for β-sheets^54^. It was prominent in the cells transfected with the control fluorescent protein, but not in the cells transfected with the fusion protein. Inversely, the absence of a strong intensity at 1235–1240 cm^−1^ is typical of an α-helix^54^. At 1240 cm^−1^, we detected a significantly weaker signal and bend in the fusion protein, indicative of its comparatively higher concentration of helices^54^. As expected, the most significant intensity differences were detected for the Raman shifts associated with proteins at 752–760 cm.^1^, 1003 cm.^1^ and the amide I (1654/1655 cm^−1^), II (1580 cm^−1^) and III (1337 cm^−1^) bands. The spectral intensities at around 750 cm^−1^ and 1582 cm^−1^ have been reported for a plethora of molecules, most dominantly for the porphyrin ring in cytochrome c^55^. For lipids, we detected significant intensity differences only at 1301 cm^−1^ and 1660 cm^−1^. Since they fall within the Amide III or I region, respectively, a contribution of protein signals seems likely.

Based on the measurements of Cx43-mTagBFP2, we trained different SVMs to distinguish between cells with high and low Cx43 content. SVMs were chosen due to their versatility and wide application in RS^56,57^. The SVMs used either a coarse or medium Gaussian kernel and were either trained on a complete Raman fingerprint spectrum (571 wavenumbers) or only on the Raman shifts where the intensity difference exceeded the double standard deviation in the training data set (82 wavenumbers). The training accuracies to discern Cx43-mTagBFP2 (high content) from the control fluorescent protein (low content) (coarse: 94%, medium: 83%) resemble those of other published supervised^56^ and unsupervised^58^ classifiers. During testing, we gradually increased the samples’ level of biological diversity to evaluate the classifier’s robustness. In the first testing data set, the low-content cells expressing the fluorescent protein only were replaced by HEK293 wildtype cells, expressing neither the control fluorescent protein nor much Cx43. With a testing accuracy of over 90% the coarse SVM exceeded the medium one. This is no surprise, because the lower kernel scale value in the medium Gaussian reduces flexibility^59^ when applied to new cell lines. The second testing data set comprised only GBM cell lines not included in the training data set. This approach is unique since most previously published classifiers were used to classify the same cell lines for training and testing^60,61^. In both SVMs, we found that models trained on only the most significant Raman shifts performed more accurately and specifically than models trained on the complete spectrum. Inversely, training on the complete spectrum resulted in a higher sensitivity. For maximum accuracy, we recommend a combination of the presented classifiers. The coarse Gaussian SVM analysing the complete Raman spectrum could be used a screening tool, because it yields a high sensitivity. The overall testing accuracy of the classifiers was again in the magnitude of previously presented classifiers^62,63^.

The shortcomings of this study include the limited sample size, which, according to the central limit theorem results in a larger standard deviation. However, our experiments show a standard deviation comparable with other studies^61,64,65^. Also, our coarse Gaussian classification algorithm could distinguish cells with an overall accuracy of 79.0%, despite the greater degree of standard deviation. The biological downstream effects of introducing mTagBFP2 or Cx43 fused to it are not fully controlled in this study. However, fluorescent proteins have been extensively used for morphologic and functional experiments, and our own validation experiments suggest proper expression^28,46^. Finally, our classifiers work well for cultured cell lines, but their generalizability might be compromised by a greater degree of heterogeneity and autofluorescence in tissue samples^17^. We tried to reduce susceptibility by reducing the spectra needed for the classifier. It should be noted that the current spectra are obtained from cellular locations with high Cx43 content, while expression levels might be lower in tissue samples.

## Methods

### Cell culture

We cultured U87MG, T98G, Ln229, Ln18, HEK293 cells in DMEM supplemented with 10% fetal bovine serum (both gibco, Thermo Fisher, Schwerte, Germany) under standard cell culture conditions. GBM cell lines had been purchased previously from the ATCC.

### Plasmid cloning by restriction enzyme digest

pDest/hCx43-EGFP-N1 and mTagBFP2-lifeact-7 were donations of Robin Shaw and Michael Davidson^23,28^. We expressed plasmids in DH5alpha (New England Biolabs, Frankfurt am Main, Germany) cultivated in Terrific Borth (Roth, Karlsruhe, Germany) supplemented with 50 mg/ml Kanamycine (Roth, Karlsruhe, Germany). For the cloning restriction digest we excised eGFP with AgeI and NotI (New England Biolabs, Frankfurt am Main, Germany) and substituted it with mTagBFP2. For the control digest we used AgeI and HindIII (New England Biolabs, Frankfurt am Main, Germany). As a control, we included mTagBFP2-ER5 (a gift from Michael Davidson)^23^.

### Protein prediction

We translated the plasmid sequences into amino acids with ExPASy (Swiss Institute of Bioinformatics, Lausanne, Switzerland)^66^ and submitted them to I-TASSER^36,37^. We visualized the predictions with Discovery Studio 2021 Client (BIOVIA, Dassault Systèmes, San Diego, USA).

### Immunocytochemistry and fluroescence imaging

We cultivated samples on round cover slips (Hartenstein, Würzburg, Germany) and fixed them with 4% formaldehyde supplemented with 2 µl Vybrant CM-DiI (Thermo Fischer Scientific, Schwerte, Germany) per 500 ml. For the indirect immunofluorescence we used a polyclonal anti-Cx43 antibody (Sigma, Merck, Darmstadt, Germany) and an Alexa Fluor secondary antibody (Thermo Fisher, Schwerte, Germany). Autofluorescence imaging was conducted using the green fluroescence filter sets ZEISS 38 (Ex: 470/40, Em: 525/50) and ZEISS 44 (Ex: 475/40, Em: 530/50). We imaged cells on an Axioplan microscope (Zeiss, Jena, Germany) equipped with an HBO 100 lamp and a monochrome camera (Axiocam 503 mono, Zeiss, Jena, Germany; 2.8 megapixel). For processing we required ZEN lite (Zeiss, Jena, Germany, 3.8 software) and Image J 1.53q (National Institutes of Health, USA)^67^. Confocal images were obtained on an Olympus FV1000 microscope (20X/0.8 N.A. air-, 60X/1.42 N.A. oil-immersion objective, Olympus Corporation, Tokyo, Japan).

### Western immunoblot

Cells were harvested at 80–90% confluency and lysed with a RIPA buffer. Lysed samples were sonicated and centrifuged at 16.000 g at 4 °C. The supernatant was stored at −20 °C until a Bradford protein assay with dye reagent (Bio-Rad Hercules, CA, USA, cat. no 500006). Samples were fractioned by a 16% SDS-page gel and incubated overnight with a polyclonal anti-Cx43 and anti-Vinculin-antibody (Sigma, Merck, Darmstadt, Germany). Primary antibodies were incubated with a horse-radish-peroxidase conjugate secondary antibody and visualized with an imager for chemiluminescent 190 signals (GE Healthcare Life Science, Munich, Germany; cat. no AI680). Western blots were conducted twice in independent samples.

### Sample preparation and Raman spectroscopy

We trypsinzed cells, fixed them in 4% formaldehyde, and washed with them with distilled water prior to plating on a polished metal slide steel slide. We used fluorescence imaging to identify Cx43-expressing regions before performing Raman spectroscopy. For Raman microscopy, we used a WITec alpha300 R (WITec GmbH, Ulm, Germany) equipped with an SHG Nd: YAG laser (532 nm, max. 22.5 mW) and a lens-based spectrometer with a CCD-camera (1024 × 128 pixel, Peltier cooled to −65 °C). The nominal spectral resolution was approx. 3 cm^−1^ per CCD pixel (600 mm^−1^ grating). Prior to each measurement session, the system was calibrated using the Silicon peak at 520.4 cm^−1^. A 50x objective was used for experiments. Using the 50 μm core of a multimode fibre we achieved a focal depth of approx. 1 μm. The integration time was typically set to 30 s per spectrum, and the laser intensity was adjusted manually for each sample to avoid the induction of damage by burning. Experiments were conducted a minimum of three times.

### Raman spectrum analysis

We processed spectra in Matlab R2023a version 9.14.0. and R2021a version 9.10.0 (The MathWorks Inc., Natick, Massachusetts) and imported them with the WITio toolbox version 2.0.1^68^. For pre-processing we baselined the fingerprint region (400–1800 cm^−1^) using an asymmetric least squares algorithm^69^. Cosmic rays were identified as sharp peaks greater than the Amide I intensity and removed. Each spectrum was vector normalized to the greatest spectral intensity.

Originally, we collected 459 spectra of the Cx43-mTagBFP2 cells. To match the number of spectra obtained from mTagBFP2 an equal number of spectra of the fusion protein (*n* = 95) were randomly chosen. Peaks were identified using the findpeaks-function of the signal processing toolbox. The difference in intensity was calculated as the absolute value of the spectral difference. Significance tests were performed with the ranksum-function for a Mann-Whitney U-Test for independent samples, choosing a p-value of 0.05 a priori. The effect sized was calculated with the computeCohen_d(x1, x2, varargin)-function in Matlab^70^. Box plots were computed using the Alternative Box Plot Toolbox version 3.2.1.0.

### Machine learning classification

For training a binary SVM data were loaded into the Matlab Classification Learner App. 80% of the imported data were used for training; 20% were held out for a five-fold cross validation. An equal number of spectra was used for each class to avoid class imbalance. In the first round, we used all 571 wavenumbers; in the second round, we only used those Raman shifts, where the intensity exceeded the double standard deviation (*n* = 82). For the medium Gaussian kernel, the scale was set to the square root of the number of predictors; for the coarse Gaussian kernel to the four-fold of the square root. The overall accuracy was calculated as the percentage of correctly classified spectra. Results were visualized as a confusion matrix and ROC-curve. The confusion matrix yielded the sensitivity (TP/(TP + FN)) and specificity (TN/(TN + FP)), where T and F denote true and false, respectively and P and N denote positive and negative.

## Electronic supplementary material

Below is the link to the electronic supplementary material.

Supplementary Material 1

## Data Availability

Matlab code and the minimal, preprocessed data set are available upon reasonable request from the corresponding author Johannes Reifenrath.

## Abbreviations

RS: Raman spectroscopy
FGRS: Fluorescence Guided Raman Spectroscopy
Cx43: Connexin 43
mTagBFP2: mTag blue fluorescent protein 2
GFP: Green fluorescent protein
ER: Endoplasmic reticulum
Cx43-mTagBFP2: Cx43 tagged with mTagBFP2
GBM: Glioblastoma multiforme
eGFP: Enhanced green fluorescent protein
SVM: Support vector machine
CMV: Cytomegalovirus
ROC: Random operator characteristic
TAE: Tris acetate EDTA buffer
TBE: Tris borate EDTA buffer
