# Supplementary material for: Fluorescence Guided Raman Spectroscopy enables the training of robust support vector machines for the detection of tumour marker proteins
Source: Sci Rep. 2025 Jul 3;15:23711. doi: 10.1038/s41598-025-08425-0 (PMC12229507; doi:10.1038/s41598-025-08425-0)
Supplement: Supplementary file 1 — Supplementary Material 1 [file 41598_2025_8425_MOESM1_ESM.pdf]

Supplementary Material

Supplementary Material S1

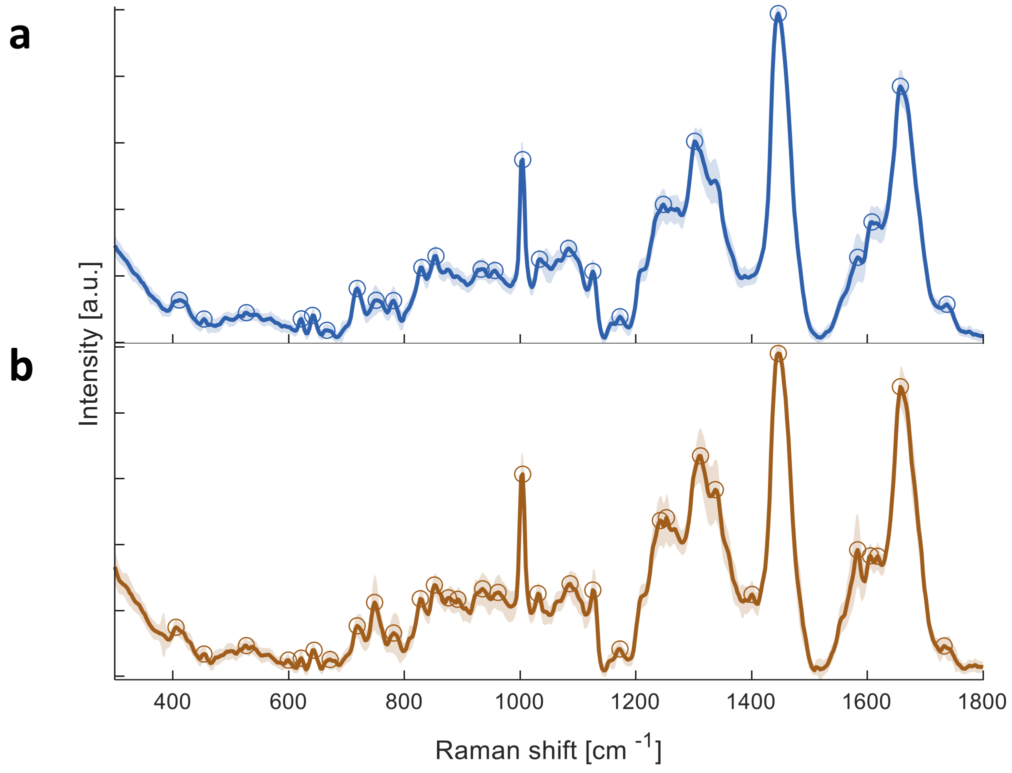

Supplementary Material S1. **Peak Location.** Peaks with a significance level p≥ 0.01 are encircled. (**a**) Averaged Raman spectrum of Cx43-mTagBFP2 (**b**) Averaged Raman spectrum of TagBFP2-ER5

Supplementary Material S2

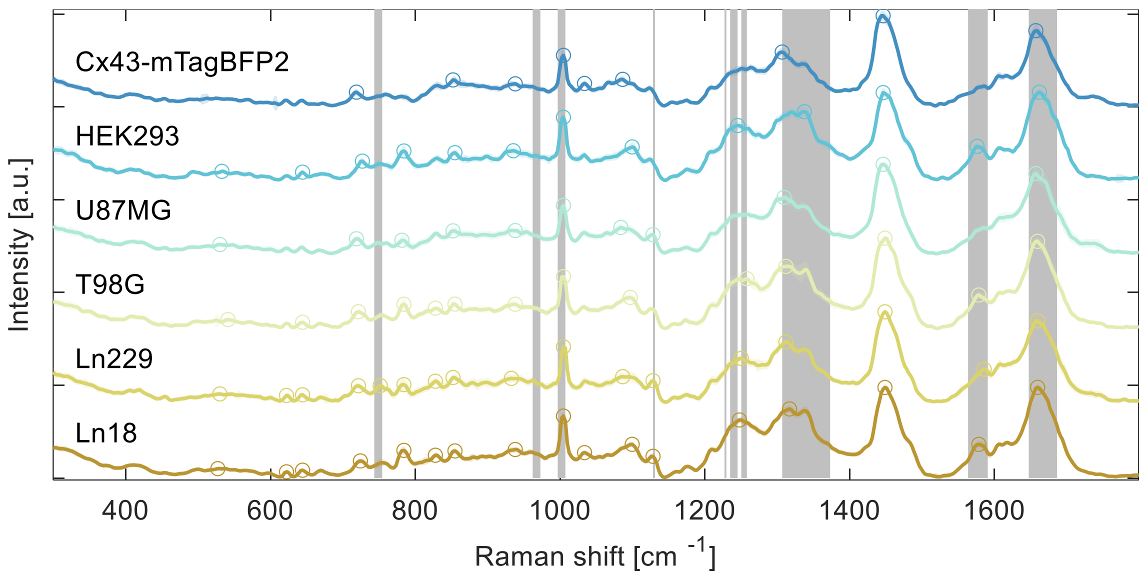

Supplementary Material S2. **Peak identification in GBM wildtype cell lines and Cx43-mTagBFP2.** Average spectra (solid line) and first standard deviation (shade) for the indicated cell lines. Raman shifts where the difference exceeds the double standard deviation are shaded in light grey. Peaks were identified by a prominence-value of 0.05.

Supplementary Material S3

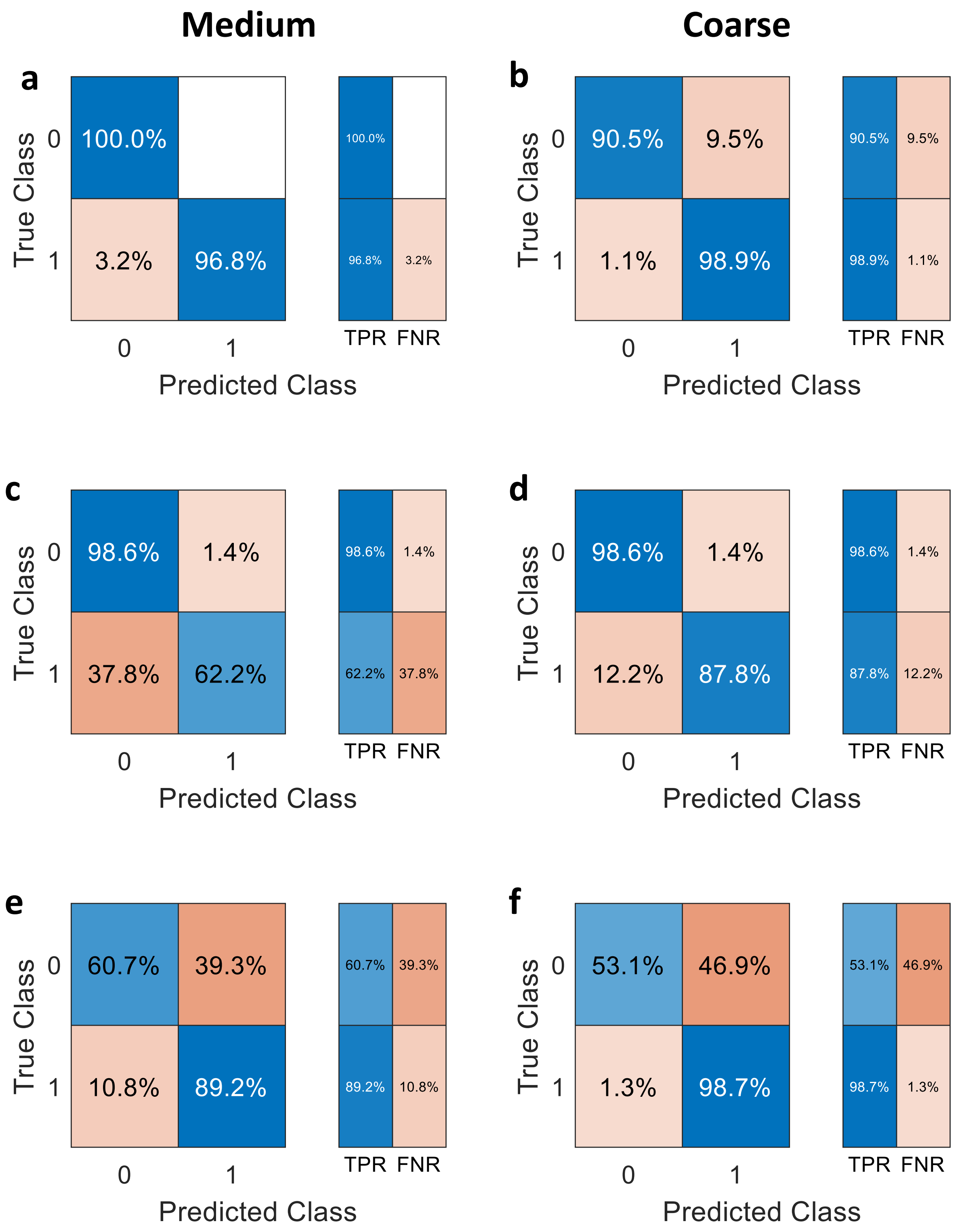

Supplementary Material S3. **Confusion Matrices for SVMs trained on all Raman shifts.** 0: low Cx43-content. 1: high Cx43-content. TPR: true positive rate. FNR: false negative rate. (**a,c,d**) Medium Gaussian SVM. (**b,d,f**) Coarse Gaussian SVM. (**a,b**) Training. (**c,d**) Testing the SVMs by Distinguishing HEK-Cx43-mTagBFP2 from Wildtype HEK293-cells. (**e,f**) Testing the SVMs on Four Human Glioblastoma Wildtype Cell Lines.

Supplementary Material S4

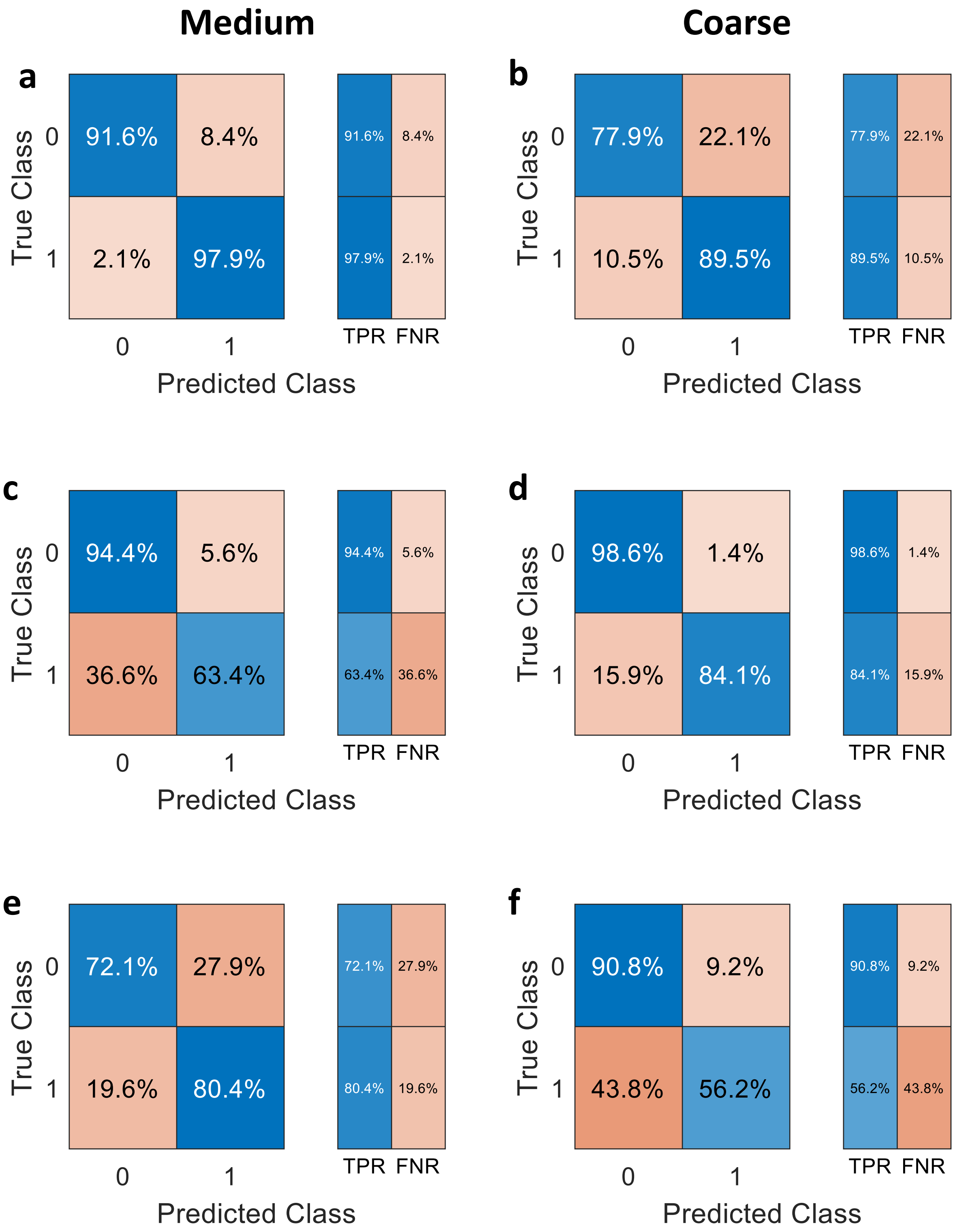

Supplemenatry Material S4. **Confusion Matrices for SVMs trained on the most important Raman shifts where the spectral intensity exceeded the double standard deviation**. 0: low Cx43-content. 1: high Cx43-content. TPR: true positive rate. FNR: false negative rate. (a,c,d) Medium Gaussian SVM. (b,d,f) Coarse Gaussian SVM. (a,b) Training. (c,d) Testing the SVMs by Distinguishing HEK-Cx43-mTagBFP2 from Wildtype HEK293-cells. (e,f) Testing the SVMs on Four Human Glioblastoma Wildtype Cell Lines.

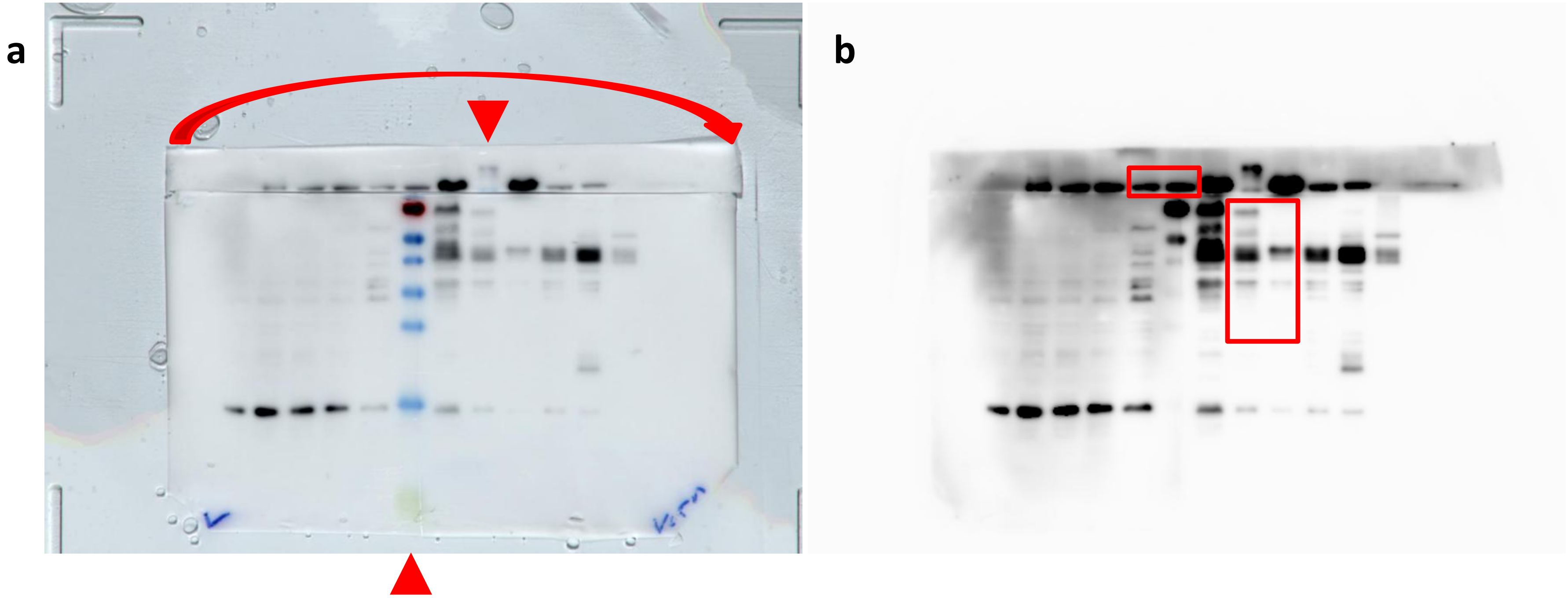

Uncropped Blot for Figure 1i. The top and bottom parts of the membrane were misaligned during imaging, as indicated by the page ruler. For realignment, the top part needs to be rotated by 180° around its short axis (arrow in a). Exposure time: 40 sec. (**a**) RGB image. Arrowheads point at the misaligned page ruler. The red rectangle indicates the cropped area used for figure 1i lane 1 (page ruler) (**b**) B/W image. The red rectangle indicates the cropped area used for figure1(i) lanes 2,3

Supplementary Material S6

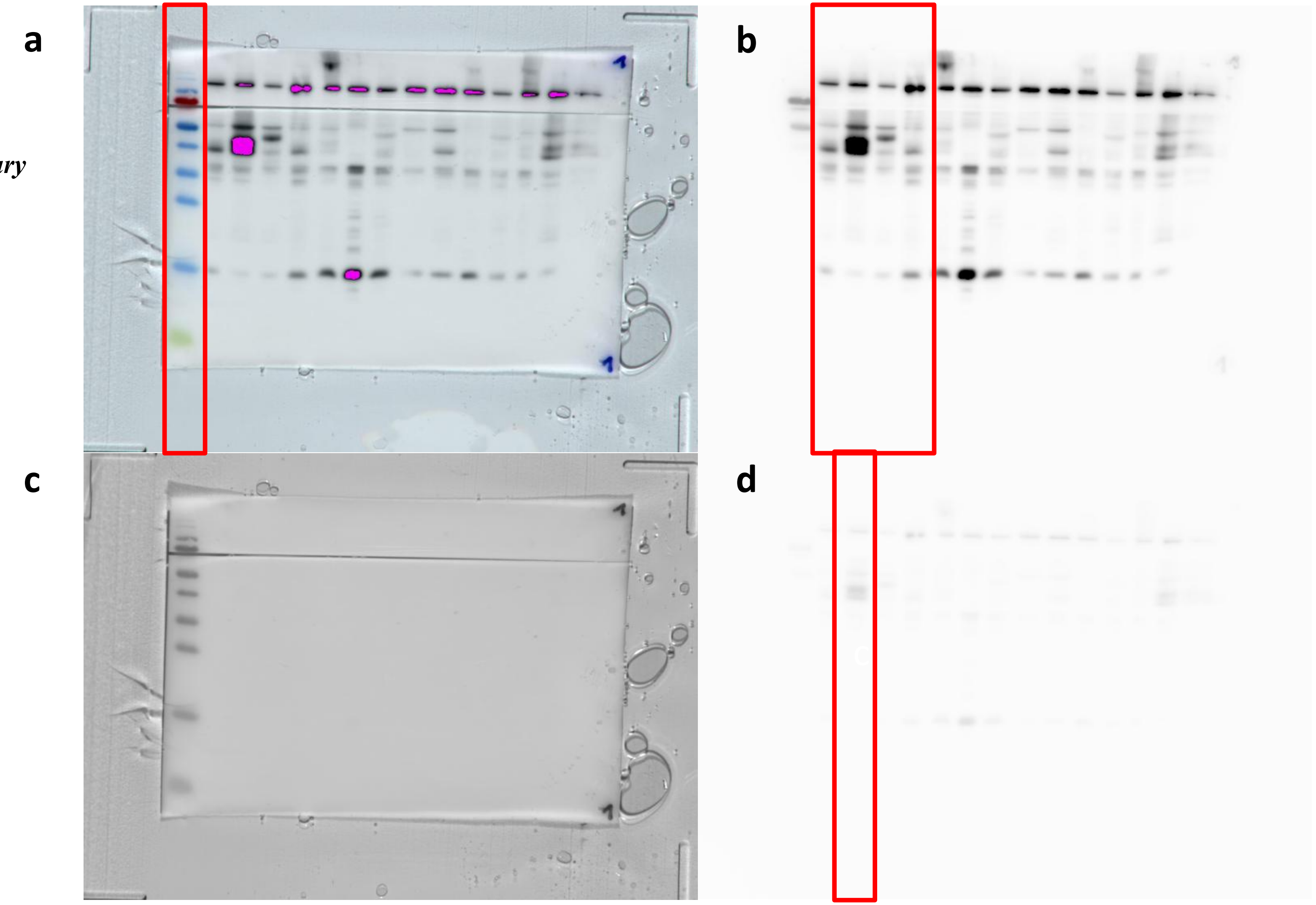

Uncropped Blot for Figure 4b. (**a,b**) exposure time: 3 min. (**a**) RGB image. The red rectangle indicates the cropped area used for figure 4b lane 1 (**b**) B/W image. The red rectangle indicates the cropped area used for figure 4b lanes 2-5 (**c,d**) exposure time: 7.5 sec. (**c**) grayscale image. (**d**) B/W image. The red rectangle indicates the cropped area used for figure 4b lane 6
